# Digital Artifacts in Education: How Design Properties Are Associated with Self-Regulated Learning, Participation, and Performance

**DOI:** 10.3390/bs16091633

**Published:** 2026-09-13

**Authors:** Ahmad Almufarreh

**Affiliations:** Department of Educational Sciences, College of Arts and Humanities, Jazan University, Jazan 45142, Saudi Arabia; almufarreh@jazanu.edu.sa

**Keywords:** artificial intelligence, digital artifacts, semiotic mediation, self-regulated learning, student participation, individual innovativeness, learning continuance, learner performance

## Abstract

Digital artifacts are preferred by both students and instructors to achieve various educational goals; yet the understanding of how digital artifacts and their properties are associated with academic development remains insufficient. Such understanding is also important as the design, modification, and use of digital artifacts, such as infographics, videos and illustrations, support both learners and instructors. Thus, drawing on the theory of semiotic mediation, this study examines how three core properties of digital artifacts, i.e., design, elaborateness, and semantic ability, are associated with learner outcomes through self-regulated learning, student participation, individual innovativeness, and learning continuance. Digital artifacts are conceptualized broadly to include conventional digital resources. Data were collected from 350 university students in Saudi Arabia and analyzed using Partial Least Squares Structural Equation Modeling (PLS-SEM). The findings show that design, elaborateness, and semantic ability are positively associated with both self-regulated learning and student participation. Such understanding, in turn, is associated with individual innovativeness, which is positively associated with learning continuance and learner performance. The results suggest that the educational value of digital artifacts depends not only on their technological availability but also on how their design characteristics support learners’ regulation, participation, experimentation, and sustained engagement. The study extends the application of semiotic mediation theory to contemporary digital learning environments and provides a behavioral explanation of how digital artifact properties are associated with academic development.

## 1. Introduction

In the current age, where technology is increasingly embedded in education, learner performance has become an important area of concern (Poupard et al., 2025; Soderstrom & Bjork, 2015). A substantial body of literature has emphasized the importance of learner performance (Deng et al., 2025) because of its association with positive learning outcomes, including relatively permanent changes in behavior and knowledge (Boakes, 2021; Garcia-Cabot et al., 2015; Schmidt & Wrisberg, 2008). Due to its nature (Kantak & Winstein, 2012; Soderstrom & Bjork, 2015), learner performance is also important from a policy perspective, as it enables educators and administrators to assess the different educational interventions (Johnsen, 2008). Furthermore, consistently high learner performance can be viewed as an indicator of the effectiveness of teaching and the quality of educational outcomes (Poupard et al., 2025). Therefore, policymakers continuously seek to improve learner performance by enhancing educational inputs, particularly those related to teaching quality, resources, and learning environments (Tan et al., 2025). In this context, the use of technology-related elements as educational inputs has increased significantly. However, despite their growing adoption, an important concern remains regarding the extent to which technology can be associated with the learner’s performance.

Before discussing technology-related elements, it is important to consider some of the key determinants of learner performance identified in existing literature. One important determinant is self-regulated learning (Zimmerman & Schunk, 2011). According to Zimmerman (2002), self-regulated learning is associated with higher levels of student performance. In this regard, Zimmerman (2002) defined self-regulated learning as “self-generated thoughts, feelings, and behaviors that are oriented to attaining goals” (Zimmerman, 2000). Another important determinant is student participation. As argued by Bekkering and Ward (2020), “active participation in class improved subjective and objective student performance.” Student participation can broadly be defined as students’ active involvement in classroom-related activities and decisions that can be associated with the direction of academic and non-academic learning activities. Overall, both self-regulated learning and active participation have emerged as important non-technological determinants of learner performance (Bekkering & Ward, 2020; Zimmerman & Schunk, 2011).

As self-regulated learning and student participation increasingly emerge as important mediators in the relationship between technology and learner performance, a key question concerns the types of technologies implemented as educational interventions. Existing literature has examined various technological approaches, including e-learning systems (Edumadze & Govender, 2024), game-based educational technologies (Bachiri et al., 2023), and other digital learning environments. Among these, digital artifacts have gained increasing prominence as an important class of intervention tools (Baccaglini-Frank et al., 2025). Digital artifacts are conceptualized by Kallinikos et al. (2013) as digital objects that are “*embedded in wider and constantly shifting ecosystems such that they become increasingly editable, interactive, reprogrammable, and distributable*.” Owing to these characteristics, digital artifacts have been widely adopted in educational contexts to support learning outcomes, particularly in areas such as mathematics education (Baccaglini-Frank et al., 2025) and project-based learning environments (Erstad, 2002). Their use has also increased in recent years with the emergence of LLMs, which have increased their accessibility, enabling learners not only to use digital artifacts but also to design, modify, and adapt them according to their learning needs. In this study, based on the assertion of Kallinikos et al. (2013), we conceptualize it as educational digital content designed to communicate information through visual and audiovisual formats, including infographics, videos, and digital media illustrations. This conceptualization distinguishes content-based digital artifacts from interactive learning platforms, editable digital resources, adaptive technologies, and AI-enabled tools.

The effectiveness of digital artifacts as educational objects associated with student learning can be understood through the theory of semiotic mediation (Mertz, 2013). The theory proposes that learners enhance their understanding through the use and design of artifacts that support problem-solving and task completion (Bussi & Mariotti, 2008). From this perspective, three key properties, i.e., design, semantic ability, and elaborateness, can determine the extent to which digital artifacts function as effective semiotic mediating tools. First, the design of an artifact should support clarity, coherence, and effective utilization (Ekbia, 2009). Second, elaborateness refers to the richness and structure of the representations provided by digital artifacts, which can support deeper engagement with learning tasks and problem-solving processes (Stuermer et al., 2017). Finally, semantic ability reflects the extent to which an artifact enables meaningful interpretation and the construction of conceptual connections during learning activities (Krippendorff & Butter, 2008). Together, these properties help ensure that learners are not only interacting with well-designed digital artifacts but are also able to derive meaningful understanding from them while completing learning tasks.

In this regard, the present study aims to address gaps in the existing literature on digital artifacts in educational contexts, as the use of artifacts has increased in the age of LLMs. It is also pertinent to clarify that the aim of this study is to examine the changing nature and accessibility of digital artifacts, rather than the use of LLMs as tools through which students design, modify, or interact with such artifacts. The core argument in the present study concerns the increasing accessibility and democratization of digital artifacts in the LLM era, rather than the specific processes through which students use LLMs to create or modify them.

Drawing on the theory of semiotic mediation, this study examines the association of digital artifact design, elaborateness, and semantic ability on self-regulated learning and student participation. This research has attempted to study the design, elaborateness, and semantic ability of digital artifacts that are static in nature, such as infographics, videos, and digital media illustrations. However, in the current era, LLMs have enabled students and instructors alike to access, design, use, and modify such static artifacts without significant financial or technical investment. Our study further investigates how these learning processes are associated with individual innovativeness and learning continuance, and ultimately to learner performance. By examining these relationships within a single integrated model, the study seeks to provide a clearer understanding of the behavioral pathways through which digital artifacts may be associated with academic development.

In addition to contributing to the literature on digital artifacts, the current study adopts a behavioral perspective on learner performance. Rather than treating digital artifact properties, self-regulated learning, student participation, individual innovativeness, and learning continuance as isolated factors, the proposed model examines how these elements operate through interconnected pathways associated with learning outcomes. This perspective is particularly relevant in contemporary digital learning environments, where the effectiveness of technological tools increasingly depends on the behavioral processes they support rather than on technological features alone.

## 2. Literature Review

### 2.1. Digital Artifacts and the Theory of Semiotic Mediation

Artifacts can be defined as objects, tools, ornaments, or modifications that are distinguished from naturally occurring objects (Merriam-Webster, 2026). Historically, artifacts have been studied to understand the culture and history of individuals and societies (Dipert, 1995). More recently, the term digital artifacts has been increasingly used across online gaming, data science, software development, and other technology-related domains to describe digitally created or mediated objects used to support particular activities (Gulotta et al., 2013). The concept of digital artifacts can be traced to the seminal work of March and Smith (March & Smith, 1995), who emphasized that technology is practical and useful because it is embodied in implements or artifacts, rather than existing only at a conceptual level. They further argued that information technology research is concerned with artificial rather than natural phenomena, focusing on human creations such as organizations and information systems. From this perspective, a digital artifact can be understood as a digitally created or technologically embodied object designed to support, mediate, or enhance human activities, interactions, and problem-solving within a particular context. This conceptualization has also been developed in subsequent studies, including the work of Orlikowski and Iacono (2001) and Kallinikos et al. (2013).

The theory of semiotic mediation is rooted in the work of Lev Vygotsky and emphasizes the role of cultural tools and signs in shaping learning and meaning-making (Mertz, 2013; Vygotsky & Leontiev, 1971). From this perspective, learning is mediated through interaction with symbolic and material tools that help individuals interpret information, construct meaning, and develop understanding (Mertz, 2013). In digital learning environments, digital artifacts can function as such mediating tools by supporting interaction with representations, symbols, and learning tasks (Morgan & Kynigos, 2014). Their effectiveness as semiotic mediators may depend on several key properties. First, design coherence can ensure that an artifact is clear, usable, and pedagogically aligned (Ekbia, 2009). Second, elaborateness can provide rich and structured representations that support deeper cognitive engagement (Stuermer et al., 2017). Third, semantic ability can enable learners to interpret information meaningfully and develop conceptual connections (Krippendorff & Butter, 2008). Together, these properties may determine how effectively digital artifacts support meaning-making and learning in contemporary digital environments, including those that involve AI-enabled tools.

Finally, an important aspect that warrants consideration concerns the potential consequences associated with the extensive use of digital tools, including digital artifacts and artificial intelligence technologies (Morgan & Kynigos, 2014). Although semiotic mediation has traditionally provided a useful theoretical lens for explaining how such tools facilitate learning, emerging research has also raised concerns regarding their excessive use and overreliance (Morgan & Kynigos, 2014). In particular, extensive reliance on digital tools may contribute to cognitive overload and emerging phenomena such as AI fatigue (Lau et al., 2026). Thus, while digital artifacts and AI technologies can facilitate learning processes and enhance performance by mediating access to information and supporting cognitive activities, excessive dependence on these tools may produce unintended negative consequences (Mertz, 2013). Overreliance may reduce learners’ active cognitive engagement, encourage passive consumption of information, and potentially undermine opportunities for independent reflection, critical thinking, and knowledge construction (Lau et al., 2026). Therefore, it is important to adopt a more balanced perspective that recognizes both the enabling role of digital artifacts and AI technologies in supporting learning and the potential cognitive consequences associated with their excessive or uncritical use.

Finally, in an era where digital tools, including digital artifacts and artificial intelligence, are increasingly embedded in educational environments, both technology-related fatigue, such as AI fatigue identified by Lau et al. (2026), and cognitive load warrant greater attention. Excessive exposure to digital tools and information may increase mental demands and fatigue, potentially inhibiting learners’ ability to regulate their learning effectively and sustain engagement (Alwahaishi & Ahmed, 2026). Reduced self-regulation and engagement may, in turn, negatively affect learning continuance and academic performance (Alwahaishi & Ahmed, 2026). Future research should therefore examine these factors to better understand the potential challenges associated with increasingly digitalized learning environments.

### 2.2. Design

The design refers to aesthetic properties of objects, such as digital artifacts, which enable users to interact with it (Folkmann, 2010). As researchers argued “especially in non-professional contexts, when design artifacts are noticed and appreciated, it is more often for their aesthetic qualities than their practical or functional ability to solve more or less complex or well-defined problems” (Folkmann, 2010). It can therefore be considered a foundational property of digital artifacts that they can be associated with both their creation and effective use in digital learning environments (Engholm, 2010). From the perspective of semiotic mediation, design plays an important role in shaping how learners interact with, interpret, and use digital artifacts to complete learning tasks (Deni & Zingale, 2017). Key characteristics of well-designed digital artifacts include clear organization, coherence, intuitive structure, and ease of navigation (Blijlevens et al., 2017). These characteristics are important because they can reduce unnecessary cognitive load and enable learners to focus more effectively on activities such as problem-solving and knowledge construction (Mazza, 2017). Accordingly, design can be viewed as an enabling condition that supports the effective use of digital artifacts and may strengthen both self-regulated learning (Alvarez et al., 2022) and active student participation (Velásquez et al., 2025).

### 2.3. Elaborateness

Elaborateness can be defined as the richness and depth of the representations embedded within digital artifacts (Pragst et al., 2018). In the context of semiotic mediation, representation is a fundamental property because it shapes how learners engage with and interpret the information conveyed through an artifact (Fadeev, 2019). Digital artifacts with a high degree of elaborateness can provide multiple layers of information, examples, and structured representations that support deeper cognitive engagement with complex concepts and learning tasks (Eysenck, 2014; Stuermer et al., 2017). Such richness may enable learners to explore, compare, and refine ideas, thereby supporting more meaningful interaction with learning content (Eghbal-Azar et al., 2016). Accordingly, elaborateness can strengthen learners’ ability to regulate their learning processes and sustain active engagement in task-oriented activities (Cho, 2020).

### 2.4. Semantic Ability

Semantic ability refers to the extent to which digital artifacts support meaningful representation, interpretation, and conceptual understanding of embedded information (Riemer, 2010). The semantic ability of an artifact is reflected in how effectively it conveys meaning through symbols, representations, and other intuitive or interactive elements that align with learners’ cognitive structures (Schlesinger et al., 1995). A key purpose of digital artifact design is therefore to enable users to perceive and interpret the intended meaning of the information presented. In this context, reduced cognitive load, ease of navigation, and effective transformation of information can be considered important aspects of semantic ability (Jagatheesaperumal et al., 2023). From the perspective of semiotic mediation, semantic ability is essential because it supports the transformation of external representations into internalized understanding and knowledge construction (O’Halloran, 2023). By enabling learners to interpret and connect information more effectively, semantic ability may strengthen both self-regulated learning (Romero et al., 2019) and sustained participation in learning activities (Tracy & Jordan, 2012).

### 2.5. Self-Regulated Learning

Self-regulated learning refers to the extent to which learners proactively plan, monitor, and regulate their cognitive, motivational, and behavioral processes during learning activities (Erdogan & Senemoglu, 2016). In recent years, educators have increasingly emphasized self-regulated learning as an important capability that enables learners to manage their learning more effectively and improve academic performance (Zimmerman & Schunk, 2011). This capability has become particularly important in digital learning environments, where students are often required to take greater responsibility for organizing and monitoring their own learning activities (Yot-Domínguez & Marcelo, 2017).

Digital artifacts can provide important cognitive and functional support for self-regulated learning by helping learners access information, organize tasks, solve problems, and monitor their progress (Khalil et al., 2024). However, the effectiveness of such support may depend on the properties of the artifacts themselves. Well-designed digital artifacts can provide clear structure and usability that facilitate goal setting and self-monitoring (Nietfeld, 2017). Similarly, elaborateness can support deeper cognitive processing by offering rich and multi-layered representations of learning content (Stuermer et al., 2017). Semantic ability can further strengthen self-regulated learning by enabling learners to interpret information meaningfully and integrate new knowledge into their existing understanding (Siadaty et al., 2011). Based on these arguments, the following hypotheses are proposed:

**H1.**  *The design of digital artifacts has a positive relationship with self-regulated learning.*

**H2.**  *The elaborateness of digital artifacts has a positive relationship with self-regulated learning.*

**H3.**  *The semantic ability of digital artifacts has a positive relationship with self-regulated learning.*

### 2.6. Student Participation

Student participation refers to the extent to which learners actively engage in learning activities through behavioral, cognitive, and interactive involvement (Anderson et al., 2019). Such participation is important because it has been associated with academic performance (Ismail et al., 2023) and greater motivation to continue learning (Zhang et al., 2023). For this reason, educators and administrators often introduce a variety of in-class and out-of-class interventions to encourage student participation (Rocca, 2010). The increasing digitalization of education has created additional opportunities to support such engagement through technology-mediated learning activities (Gopinathan et al., 2022).

Digital artifacts can be associated with student participation by structuring interaction with learning content, tasks, and other learners. In this regard, the design of digital artifacts can facilitate participation by making learning activities more accessible, intuitive, and interactive (Walton et al., 2019). Similarly, elaborateness may promote sustained engagement by providing rich and multifaceted representations that encourage further exploration and collaborative use (Zahn et al., 2012). Semantic ability can also strengthen participation by enabling learners to interpret content more clearly and confidently to discussions and shared learning activities (B. Chen & Chen, 2024). Based on these arguments, the following hypotheses are proposed:

**H4.**  *The design of digital artifacts has a positive relationship with student participation.*

**H5.**  *The elaborateness of digital artifacts has a positive relationship with student participation.*

**H6.**  *The semantic ability of digital artifacts has a positive relationship with student participation.*

### 2.7. Individual Innovativeness

Individual innovativeness, within the educational context, refers to a learner’s tendency to explore, adopt, and apply new ideas, methods, and approaches to support learning and improve academic performance (Wang & Lin, 2021). The increasing use of digital technologies in education has created greater opportunities for students to develop such innovativeness by experimenting with new tools, resources, and ways of solving problems (Devisakti & Muftahu, 2023). Digital artifacts, in particular, can provide flexible environments in which learners explore alternatives, modify existing resources, and develop novel approaches to learning tasks (Shaikh et al., 2026). Thus, based on this assertion, which is also adopted by (Wang & Lin, 2021), student innovativeness is an individual capability whose outcomes depend on its level (high and low). By adopting such a view, it can be argued that development of innovativeness as a capability depends on students’ engagement and learning (self-regulated) (Melamed & Shauman, 2020).

The study by Melamed and Shauman (2020) provides an interesting insight with regard to the contextual dependencies of innovativeness. Based on action research findings, students who actively engage in project-based tasks, solve real-world problems, and learn through experimentation demonstrate significantly higher levels of innovation. This suggests that hands-on involvement and experiential learning are key drivers of innovative thinking and creativity (Melamed & Shauman, 2020). Thus, it can be argued that self-regulated learning can encourage students to experiment with alternative strategies, reflect on their progress, and adapt their approaches to different learning situations, thereby strengthening innovative learning behaviors (Schuitema et al., 2012). Similarly, active student participation can promote interaction, collaboration, and knowledge exchange, which may stimulate creative thinking and the development of new learning approaches thereby enhancing innovativeness as a capability (Acar & Tuncdogan, 2019).

Further, based on the assertions of Innovation Diffusion Theory, enhanced innovativeness as a capability can generate several important outcomes. One such outcome, which has been empirically established in the existing literature, is continuance intention (Ng, 2023). Learning continuance is both a desirable and necessary outcome in the current educational era (Fischer et al., 2023). Therefore, fostering such behavior through innovativeness can offer significant theoretical and practical value. Furthermore, performance has emerged as another important outcome of innovativeness (Wang & Lin, 2021), as students’ use of this capability enables them to address learning-related problems more efficiently and effectively. More importantly, innovativeness may be associated not only with student performance but also with learning continuance, which may be related to academic performance (Melamed & Shauman, 2020). Based on these arguments, the following hypotheses are proposed:

**H7.**  *Self-regulated learning has a positive relationship with individual innovativeness.*

**H8.**  *Student participation has a positive relationship with individual innovativeness.*

### 2.8. Learning Continuance

Learning continuance refers to learners’ intention to persist in and continue using particular digital artifacts or learning approaches over time (Bhattacherjee, 2001; Nabavi et al., 2016). In contemporary digital learning environments, sustained engagement with digital tools and resources can play an important role in supporting continued learning and academic development (Ranjan & Mukherjee, 2026). However, such continuance may depend on learners’ willingness to explore and adapt to new learning experiences. Further, due to the emergence of and heavy reliance on these digital tools, a key phenomenon of fatigue has been observed, which can affect various behavioral outcomes such as continuance (Lau et al., 2026).

Individual innovativeness can be associated with learning continuance by encouraging students to experiment with new tools, approaches, and digital resources and to recognize their potential value for learning (H.-J. Chen, 2025). Learners with higher levels of innovativeness may therefore be more likely to perceive digital learning environments as useful, engaging, and adaptable to their evolving needs, which can strengthen their intention to continue using them (Pekel et al., 2022). Digital artifacts can further support this process by providing opportunities for exploration, experimentation, and engagement with new forms of learning (Hong et al., 2017). Based on these arguments, the following hypothesis is proposed:

**H9.**  *Individual innovativeness has a positive relationship with learning continuance.*

### 2.9. Learner Performance

Learner performance has been defined as performance towards learning and goals orientation using engagement, persistence, effort, and goal-directed behaviors in pursuing academic outcomes (Dweck & Leggett, 1988). It encompasses how learners respond to challenging academic tasks, sustain effort following setbacks, adapt to difficult circumstances, and remain committed to achieving academic goals (Kantak & Winstein, 2012; Soderstrom & Bjork, 2015). In this sense, learner performance reflects not only academic achievement but also the behavioral and motivational processes (Roedel et al., 1994) through which students pursue successful learning outcomes (Boakes, 2021; Johnsen, 2008).

The increasing integration of digital technologies into education has created new opportunities to support learner performance through innovative tools and resources (Garcia-Cabot et al., 2015). Digital artifacts can be associated with this process by helping learners access information, represent complex concepts, and engage with learning activities in more flexible and interactive ways (Maurya & Singh, 2026). However, their effectiveness may depend on learners’ willingness to explore, adapt, and use these resources meaningfully. Individual innovativeness may therefore enhance learner performance by encouraging students to experiment with digital tools, adopt new learning strategies, and apply alternative approaches to problem-solving (Devisakti & Muftahu, 2023; Pan et al., 2024). Similarly, learning continuance may strengthen performance by supporting sustained engagement with learning resources and allowing learners to develop knowledge and skills over time (Panigrahi et al., 2018). Based on these arguments, the following hypotheses are proposed:

**H10.**  *Individual innovativeness is positively associated with learner performance.*

**H11.**  *Learning continuance is positively associated with learner performance.*

## 3. Materials and Methods

The present study empirically tests a conceptual model examining how key properties of digital artifacts, i.e., design, elaborateness, and semantic ability, can be associated with learner performance. In doing so, the study investigates the roles of self-regulated learning, student participation, individual innovativeness, and learning continuance with which digital artifact properties are associated. A quantitative research design was employed (Fischer et al., 2023), using a structured survey for data collection and Partial Least Squares Structural Equation Modeling (PLS-SEM) as the primary data analysis technique.

### 3.1. Research Design

**Figure 1:**
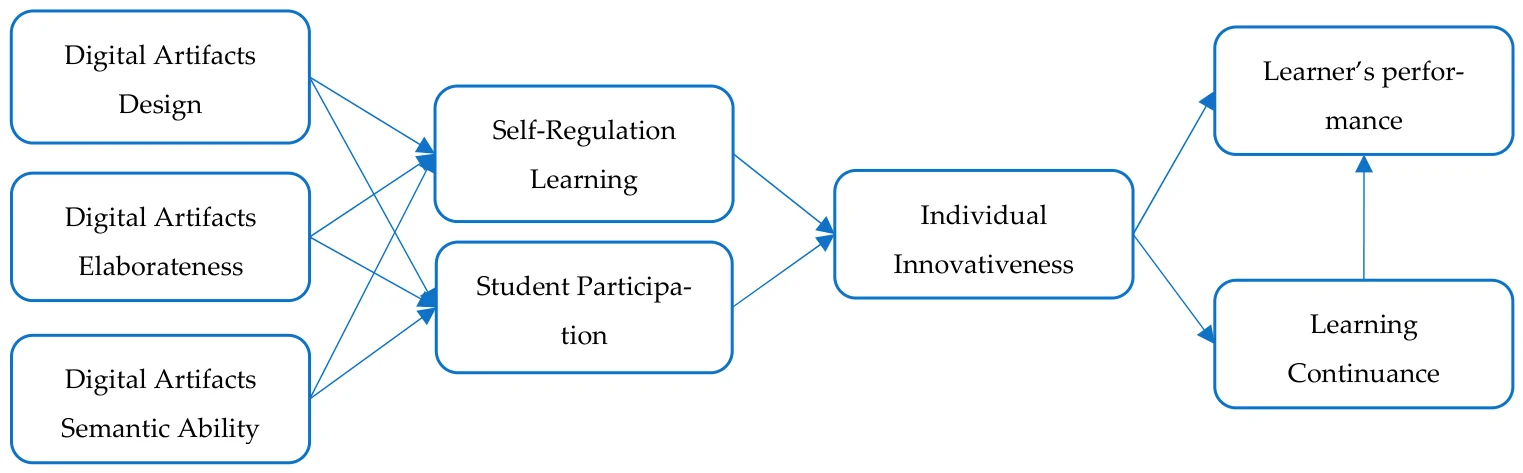
Conceptual framework.

The present study aims to validate a conceptual framework grounded in the theory of semiotic mediation and to examine how digital artifact properties associated with learning processes and outcomes (Fischer et al., 2023). Specifically, the framework investigates the relationship of digital artifact design, elaborateness, and semantic ability with self-regulated learning and student participation, and how these factors are subsequently associated with the individual innovativeness, learning continuance, and learner performance, as illustrated in Figure 1. To examine these relationships, the study employed a quantitative research design, which enabled the collection and analysis of empirical data in relation to the proposed hypotheses (Nardi, 2018).

### 3.2. Data Collection Instrument

Data were collected using a structured questionnaire comprising five-point Likert-scale items adapted from previously validated measurement instruments (Table 1). Digital artifact design was measured using the scale of Blijlevens et al. (2017), capturing the clarity, coherence, organization, integration, and distinctiveness of artifact design. Digital artifact elaborateness was measured using Reynolds (1997), assessing the richness, depth, and cognitive engagement generated by the artifact. Digital artifact semantic ability was measured using DeLone and McLean (2003), capturing the extent to which artifacts provide clear, meaningful, relevant, and understandable information. Self-regulated learning was measured using Erdogan and Senemoglu (2016), assessing learners’ planning, monitoring, regulation, and management of learning activities, while student participation was measured using Anderson et al. (2019), capturing students’ involvement, collaboration, and opportunity to express their views in learning and university activities. Individual innovativeness was measured using Leavitt and Walton (1975), reflecting learners’ willingness to explore, adopt, and experiment with new ideas and approaches. Learning continuance was measured using Bhattacherjee (2001), assessing learners’ intention to continue using digital learning artifacts and tools. Finally, learner performance was measured using Roedel et al. (1994), capturing learners’ persistence, effort, motivation, goal orientation, and responses to challenging academic tasks. The survey captured participants’ accumulated perceptions of the properties of digital artifacts and other key constructs developed and experienced through their prior coursework. These prior experiences provided the basis for participants’ evaluations of the digital learning environment. The complete measurement items are presented in Appendix A.

### 3.3. Population and Sampling

The target population of this study consisted of university students in the Kingdom of Saudi Arabia. University students were selected because of their extensive use of digital artifacts for learning purposes and their opportunities to use, design, modify, explore, and experiment with such artifacts in academic contexts. The study employed a non-probability purposive sampling technique to recruit students who had experience using, designing, or modifying digital artifacts for learning-related purposes (Etikan et al., 2016). The sample of the current study consists of 64% male and 36% female respondents, with an average age of 24.5 years and 3.9 years of professional work experience. Similarly, on average, the study respondents possessed at least an undergraduate degree. A total of 350 valid responses were collected. This sample size was considered adequate for the study objectives and appropriate for conducting analysis of the proposed model (Saunders et al., 2012). The present study distributed 500 questionnaires to students, of which 412 responses were received, yielding a response rate of 82.4%. After excluding incomplete and inappropriate responses, 344 usable responses were retained. To achieve the desired sample size of 350, six additional respondents were recruited.

The purposive sampling approach also enabled the study to focus on respondents with relevant experience of digital artifacts, thereby ensuring that participants were able to evaluate the constructs examined in the study.

### 3.4. Techniques of Data Analysis

Partial Least Squares Structural Equation Modeling (PLS-SEM) was employed as the primary statistical technique to test the proposed hypotheses and validate the conceptual framework grounded in the theory of semiotic mediation (Hair et al., 2019). The analysis involved the assessment of both the measurement model and the structural model. The measurement model was evaluated to establish the reliability and validity of the constructs, whereas the structural model was examined to test the hypothesized relationships among the study variables (Hair et al., 2011). PLS-SEM was considered appropriate for this study because of its suitability for analyzing complex models involving multiple latent constructs and predictive relationships (Hair et al., 2017). In this regard, the current research has employed the statistical software of SmartPLS 4.1.1.8 (SmartPLS GmbH, Bönningstedt, Germany), which is considered state-of-the-art software for estimating the research model using PLS-SEM as an analytical technique. In addition, common method bias was assessed using Harman’s single-factor test (Aguirre-Urreta & Hu, 2019). Harman’s single-factor test was estimated using IBM-SPSS software, version 25 (IBM Corp., Armonk, NY, USA).

## 4. Results

### 4.1. Common Method Bias

Common method bias (Kock et al., 2021) was assessed using Harman’s single-factor test (Aguirre-Urreta & Hu, 2019). The analysis was conducted using IBM SPSS Statistics version 25 (IBM Corp., Armonk, NY, USA). As shown in Table 2, the first extracted factor accounted for 27.68% of the total variance, which is below the commonly used 50% threshold (Aguirre-Urreta & Hu, 2019). This result suggests that common method bias is unlikely to represent a substantial concern in the present study. However, with the help of PLS-SEM, the present study also assessed the variance inflation factor (VIF). The results, as reported in Appendix B, suggest that all item-level VIF values were below the threshold of 5.0, with the highest VIF value being 4.078. These findings provide further evidence that common method bias is unlikely to pose a serious concern in this study.

### 4.2. Descriptive Analysis of Sample

Table 3 presents the demographic characteristics of the study sample. Of the 350 respondents, 64% were male and 36% were female. In terms of age, the majority of respond-ents were between 18 and 24 years old (68.3%), followed by those aged 25–34 years (27.1%) and 35–44 years (4.6%).

Regarding educational level, 51.7% of the respondents were undergraduate students, 34.3% were postgraduate students, 8.3% were enrolled in or had completed doctoral studies, and 5.7% had completed high school. In terms of work experience, 73.1% reported 0–4 years of experience, 20.0% reported 5–10 years, and 6.9% reported 11 years or more.

The respondents represented a range of academic disciplines. Specifically, 26.3% were from Business Administration, 23.7% from Engineering and Technology, 17.1% from Social Sciences and Humanities, 16.9% from Medical and Related Sciences, and 16.0% from Data Science and Artificial Intelligence. Overall, the sample reflects participants from diverse educational backgrounds and fields of study.

### 4.3. Construct Validity and Reliability

Indicator reliability was first assessed by examining the outer loadings of the measurement items. All retained indicators demonstrated outer loadings above the recommended threshold of 0.70, with values ranging from 0.743 to 0.892, indicating satisfactory indicator reliability.

The reliability and convergent validity of the measurement model were then assessed using Cronbach’s alpha, composite reliability, and average variance extracted (AVE) (Hajjar, 2018; Li & Lay, 2024). As presented in Table 4, all constructs demonstrated satisfactory internal consistency, with Cronbach’s alpha and composite reliability values exceeding the recommended threshold of 0.70 (Hair et al., 2019; Hancock & Mueller, 2001).

Convergent validity was assessed using AVE. All constructs reported AVE values above the recommended threshold of 0.50, indicating that the measurement items adequately explained the variance of their respective constructs (Colliver et al., 2012; Hair et al., 2019). Overall, the results confirm that the measurement model achieved acceptable levels of indicator reliability, internal consistency, and convergent validity.

### 4.4. Discriminant Validity

Discriminant validity was assessed using the Heterotrait–Monotrait ratio of correlations (HTMT), which evaluates the degree to which the constructs in the model are empirically distinct from one another (Cheung et al., 2024; Voorhees et al., 2016). As presented in Table 5, all HTMT values were below the recommended threshold of 0.85, indicating adequate discriminant validity among the study constructs (Cheung et al., 2024).

These results suggest that the constructs included in the model measure conceptually distinct phenomena and that discriminant validity was established satisfactorily.

### 4.5. Explained Variance

The explanatory power of the structural model was assessed using the coefficient of determination (R^2^). As shown in Table 6, digital artifact design, elaborateness, and semantic ability explained 39.0% of the variance in self-regulated learning and 38.2% of the variance in student participation. Self-regulated learning and student participation together explained 48.8% of the variance in individual innovativeness, while individual innovativeness explained 24.7% of the variance in learning continuance. Finally, individual innovativeness and learning continuance jointly explained 47.7% of the variance in learner performance.

Overall, these results indicate that the proposed model demonstrates moderate explanatory power for the key endogenous constructs examined in the study (Ozili, 2023).

### 4.6. Model Fit

The model fit was assessed using the Standardized Root Mean Square Residual (SRMR) (Shi et al., 2020). SRMR measures the difference between the observed correlation matrix and the model-implied correlation matrix, with lower values indicating better model fit (Ximénez et al., 2022). As shown in Table 7, the estimated model produced an SRMR value of 0.038, indicating an acceptable fit between the proposed model and the observed data.

### 4.7. Effect Size (f^2^)

The present research finally assessed the effect size in the measurement model using the measure of f^2^. The results shown in Table 8 suggest that exogenous constructs have meaningful impact on endogenous constructs. Digital artifact design demonstrated a medium effect on self-regulated learning (f^2^ = 0.308) and student participation (f^2^ = 0.207), while digital artifact elaborateness showed a small-to-medium effect on self-regulated learning (f^2^ = 0.189) and a medium effect on student participations (f^2^ = 0.247). Digital artifact semantic ability demonstrated small-to-medium effects on self-regulated learning (f^2^ = 0.162) and student participations (f^2^ = 0.181). Furthermore, individual innovativeness had a medium effect on learning continuance (f^2^ = 0.328) and learning performance (f^2^ = 0.290). Learning continuance had a small-to-medium effect on learning performance (f^2^ = 0.174). Finally, both self-regulated learning (f^2^ = 0.284) and student participations (f^2^ = 0.303) demonstrated medium effects on individual innovativeness. Overall, the findings indicate that the proposed predictors make substantive contributions to explaining the variance in the endogenous constructs.

### 4.8. Structural Model

The structural model was assessed using a bootstrapping procedure with 5000 sub-samples to evaluate the significance of the proposed relationships (Hair et al., 2011; Hair et al., 2019). As presented in Table 9, all hypothesized relationships were statistically significant at *p* < 0.05, supporting H1–H11.

The results therefore provide empirical support for the proposed conceptual framework grounded in the theory of semiotic mediation. Specifically, the findings indicate that the properties of digital artifacts are correlated with learner outcomes through a sequence of association, i.e., relationship which involves self-regulated learning, student participation, individual innovativeness, and learning continuance.

### 4.9. Mediation Analysis

The mediating relationships proposed in the research model were assessed using specific indirect analysis of PLS-SE obtained through the bootstrapping procedure (Hair et al., 2019). As presented in Table 10, all proposed indirect results were statistically significant at *p* < 0.05, providing additional support for the behavioral pathways specified in the conceptual model. Further, our full serial indirect paths from digital artifact properties, i.e., design, elaborateness and semantic ability, self-regulated learning and participation, innovativeness, learning continuance, and finally learner performance, were also found to be significant (*p* < 0.05). Appendix C provides a full list of the serial mediation path tested as part of PLS-SEM analysis and necessary for drawing inferences about the paths presented in Table 10.

Specifically, self-regulated learning and student participation significantly mediated the relationships between digital artifact properties and individual innovativeness. In addition, individual innovativeness significantly mediated the relationships of self-regulated learning and student participation with learning continuance and learner performance. These findings indicate that the relationship of digital artifact properties on learner outcomes operates not only through direct relationships but also indirect relationships involving self-regulation, participation, innovativeness, and continuance.

## 5. Discussion

The increasing digitization of education has created new opportunities to enhance learner performance (Tinjić & Nordén, 2024). In response to these developments, researchers have examined various technologies and digital learning environments, including information systems (Saa et al., 2019), e-learning (Qiu et al., 2022), online courses (Phan et al., 2016), and learning management systems (Oguguo et al., 2021). These technologies and tools are often institutional in nature because their implementation typically requires substantial financial investment as well as organizational and technological infrastructure. However, learners also make extensive use of open and widely accessible digital artifacts to support and facilitate their learning (Baccaglini-Frank et al., 2025). Although digital artifacts are widely created, shared, used, and modified across learning environments, relatively little research has examined the understanding through which these can be associated with learner performance. Against this background, the present study, grounded in the theory of semiotic mediation, examines how three key properties of digital artifacts, i.e., design, elaborateness, and semantic ability, can be associated with self-regulated learning and student participation. These properties are further expected to foster individual innovativeness through self-regulated learning and student participation, while individual innovativeness and learning continuance are ultimately associated with learner performance. The findings provide empirical support for the proposed model, indicating that digital artifact properties are linked to learning processes and outcomes through self-regulated learning, student participation, individual innovativeness, and learning continuance.

### 5.1. Self-Regulated Learning and Student Participation

The present study, grounded in the theory of semiotic mediation, proposes that digital artifacts can be associated with learning through self-regulated learning (Alvarez et al., 2022) and student participation (Zhang et al., 2023). This proposition is consistent with the central premise of semiotic mediation, which emphasizes the role of tools and artifacts being associated with cognitive development (Bussi & Mariotti, 2008). For such development to occur, learners need to actively engage with these artifacts within self-directed and participatory learning processes that involve exploration, interaction, and experimentation (Alvarez et al., 2022; Zhang et al., 2023).

The study further proposed that key properties of digital artifacts, i.e., design, elaborateness, and semantic ability, are associated with both self-regulated learning and student participation. The results showed that digital artifact design was positively associated with self-regulated learning (β = 0.435, *p* < 0.001) and student participation (β = 0.359, *p* < 0.001). These findings support the argument that design of digital artifacts can provide clear structure, intuitive interaction, and cognitive support that help learners plan, monitor, and engage more effectively in learning activities (Alvarez et al., 2022; Velásquez et al., 2025).

The study also examined the role of elaborateness, defined as the richness and depth of representations embedded within digital artifacts (Pragst et al., 2018). Drawing on the theory of semiotic mediation, the study proposed that such representational depth like design can also be associated with both self-regulated learning (Cho, 2020) and student participation (Eghbal-Azar et al., 2016). Consistently, PLS-SEM results have supported such assertion where elaborateness was positively associated with self-regulated learning (β = 0.341, *p* < 0.001) and student participation (β = 0.393, *p* < 0.001). These findings suggest that rich, structured, and multi-layered representations can facilitate deeper cognitive processing and encourage more active engagement with learning activities. The association with student participation further indicates that elaborateness may be particularly important for sustaining learners’ involvement and interaction in digitally mediated environments (Cho, 2020; Morgan & Kynigos, 2014).

Finally, semantic ability was proposed to support self-regulated learning (Romero et al., 2019) and student participation (Tracy & Jordan, 2012) by helping learners interpret and connect conceptual information more effectively. The results showed that semantic ability was positively associated with self-regulated learning (β = 0.315, *p* < 0.001) and student participation (β = 0.335, *p* < 0.001). These findings indicate that semantically meaningful digital artifacts can strengthen learners’ ability to interpret, connect, and internalize knowledge, thereby supporting both self-regulatory processes and participation in learning activities (O’Halloran, 2023; Riemer, 2010; Romero et al., 2019; Tracy & Jordan, 2012).

### 5.2. Individual Innovativeness

The results of the present study indicate that both self-regulated learning and student participation are positively associated with individual innovativeness. The PLS-SEM results showed that self-regulated learning (β = 0.413, *p* < 0.001) and student participation (β = 0.427, *p* < 0.001) were both positively associated with individual innovativeness. These findings suggest that learners’ innovative capacity is associated with both their ability to regulate their own learning and their active involvement in learning activities (Melamed & Shauman, 2020; Schuitema et al., 2012). The effect of student participation further indicates that interactive and participatory learning experiences may be particularly important in encouraging innovative learning behaviors (Melamed & Shauman, 2020).

The study also examined the indirect association of digital artifact properties on individual innovativeness through self-regulated learning and student participation. The results showed that all proposed indirect paths were statistically significant (*p* < 0.001). Digital artifact design had significant indirect association on individual innovativeness through self-regulated learning (β = 0.180) and student participation (β = 0.153). Similarly, elaborateness is associated with innovativeness indirectly through self-regulated learning (β = 0.141) and student participation (β = 0.168). Semantic ability also showed significant indirect association through self-regulated learning (β = 0.130) and student participation (β = 0.143).

These findings reinforce the role of self-regulated learning and student participation through which digital artifact properties are associated with the individual innovativeness. More importantly, they provide a theoretical basis for conceptualizing innovativeness not as a static individual trait, but as a dynamic capability that can be developed and strengthened through learners’ engagement with their learning environment. From this perspective, innovativeness emerges through an ongoing process in which learners regulate their learning, actively participate in learning activities, and experiment with alternative ways of using digital artifacts. Thus, the findings suggest that the innovative capacity of students is not determined solely by their inherent propensity to innovate; rather, it can be cultivated through learning experiences and artifact-mediated interactions that stimulate exploration, experimentation, and adaptation. This extends the understanding of individual innovativeness by highlighting the behavioral and contextual conditions through which such capability develops and becomes consequential for subsequent learning outcomes.

An important argument that needs attention is the dynamic nature of innovativeness (Soxadaliyev, 2018). Defined (also in the current study) as a dynamic capability, Innovativeness remains evident in the current literature, which shows highly innovative students tend to prefer and explore the use and experimentation of technology and tools (Wang & Lin, 2021). These students, motivated by novel (learning) experience, can use a variety of tools for regulating their learning and performance (Wang & Lin, 2021). Therefore, although the current study provides evidence of an association between self-regulated learning (and student participation) and increasing innovativeness as a result of the use of digital artifacts, students’ inherent innovative capability can also lead to the exploration of technological tools for enhancing their academic performance (Şahin & Dursun, 2022; Wang & Lin, 2021).

Finally, the current research has employed a survey approach to collect the data. Such an approach is both appropriate and effective for capturing participants’ accumulated perceptions and experiences of digital artifacts. However, future research could complement these findings through experimental or quasi-experimental designs. Such studies could present participants with controlled and standardized digital artifact samples, ensuring that all participants evaluate comparable properties. This method could help minimize variation arising from differences in prior individual experiences. Experimental evidence could therefore provide stronger support for the observed relationships by isolating the effects of specific digital artifact characteristics.

### 5.3. Learning Continuance

The present study proposed that individual innovativeness is positively associated with learning continuance (Bhattacherjee, 2001; Nabavi et al., 2016). This relationship is based on the view that learners who are more willing to explore, experiment, and adopt new learning approaches are also more likely to continue learning using digital artifacts as key resources over time (H.-J. Chen, 2025; Pekel et al., 2022). In digitally mediated learning environments, such innovativeness can help learners recognize new possibilities for using technological tools and sustain their engagement with learning activities. The PLS-SEM results showed that individual innovativeness was positively associated with learning continuance (β = 0.497, *p* < 0.001). This finding suggests that learners who demonstrate higher levels of innovativeness are more likely to persist in using digital artifacts as part of their learning processes. It also indicates that continued learning may be strengthened when learners are encouraged to explore and adapt digital resources according to their evolving needs.

The indirect results further showed that self-regulated learning significantly associated with learning continuance through individual innovativeness (β = 0.205, *p* < 0.001), while student participation also had a significant indirect effect on learning continuance through individual innovativeness (β = 0.212, *p* < 0.001). These findings suggest that self-regulation and participation are associated with sustained learning alongside greater innovativeness. In other words, learners who actively regulate and participate in their learning may become more willing to experiment with digital artifacts, which in turn can strengthen their intention to continue learning with such tools. However, increasing exposure and heavy reliance on digital artifacts may also generate fatigue, potentially reducing learners’ motivation and willingness to engage with them over time (Lau et al., 2026). Thus, sustained learning may depend on balancing active engagement with digital tools and avoiding excessive reliance that could undermine continued use.

### 5.4. Learner Performance

The findings of the present study indicate that learner performance is associated with a sequence of direct and indirect association rather than innovativeness alone. Specifically, the model indicates associations between design, elaborateness, and semantic ability and self-regulated learning and student participation, which are in turn associated with individual innovativeness, learning continuance, and learner performance. This pattern highlights the importance of understanding how learners interact behaviorally with digital artifacts, rather than assuming that technological characteristics directly translate into improved academic outcomes.

The results showed that individual innovativeness was positively associated with learner performance (β = 0.449, *p* < 0.001), while learning continuance was also positively associated with learner performance (β = 0.348, *p* < 0.001). These findings suggest that learners who are more willing to explore and adopt new learning approaches, and who sustain their engagement with digital resources over time, are more likely to report learning outcomes (Devisakti & Muftahu, 2023; Panigrahi et al., 2018).

The indirect analysis provides further support for this behavioral interpretation. Self-regulated learning was significantly associated with learner performance through individual innovativeness (β = 0.185, *p* < 0.001), while student participation had a significant indirect effect on learner performance through individual innovativeness (β = 0.192, *p* < 0.001). In addition, individual innovativeness was indirectly associated with learner performance through learning continuance (β = 0.173, *p* < 0.001). Overall, these results indicate that learner performance is associated through interconnected behavioral pathways involving self-regulation, participation, innovativeness, and sustained learning engagement.

## 6. Conclusions

The educational technology literature has largely been informed by established theories such as the Technology Acceptance Model and related frameworks (Davis, 1989). These perspectives have provided important insights into how learners adopt and use technologies. However, they offer more limited explanations of the learning outcomes that emerge after adoption, particularly outcomes such as learner performance (Soderstrom & Bjork, 2015). In addition, much of this literature has focused on institutionally provided technologies, whereas learners are increasingly interacting with non-institutionally supported tools, such as digital artifacts, that they can use, modify, and adapt for their own learning purposes. Previous research has examined the educational potential of such artifacts (Baccaglini-Frank et al., 2025), but further attention is needed to build an understanding of how their artifact properties are associated with learning outcomes.

The present study addresses this gap by shifting attention from adoption toward the mediating role of technology (digital artifacts) in the learning process. Rather than treating these artifacts merely as technologies to be accepted or used, the study conceptualizes their design, elaborateness, and semantic ability as properties that are associated with self-regulated learning and student participation. The findings further highlight the associations among these behavioral processes, individual innovativeness, learning continuance, and learner performance. In this way, the study extends the application of semiotic mediation theory to contemporary digital learning environments.

The findings provide empirical support for the proposed model by showing that digital artifact properties are associated with learner performance. Design, elaborateness, and semantic ability were positively associated with self-regulated learning and student participation, which were in turn associated with individual innovativeness. Individual innovativeness was further positively associated with learning continuance and learner performance. These results highlight the importance of examining not only the characteristics of digital tools but also the behavioral processes through which learners engage with them.

In conclusion, the study underscores the importance of digital artifacts, including AI-enabled tools as part of this broader category, in understanding meaningful learning outcomes through the lens of semiotic mediation. Moving beyond technology acceptance and adoption perspectives, the findings show that the properties of digital artifacts can structure interaction and support cognitive and behavioral engagement, thereby enabling learners to regulate their learning and participate more actively in educational activities. These processes, in turn, are associated with individual innovativeness and sustained learning engagement, which are further associated with learner performance.

### 6.1. Implications for Theory

The present study offers several implications for the literature and theory. First, it extends educational technology research beyond the traditional focus on technology acceptance and actual use by examining the learning outcomes that emerge from the mediating role of digital artifacts. Rather than treating digital technologies only as systems that learners adopt, the study highlights how their properties can be associated with post-use learning processes and behavioral outcomes.

Second, the findings emphasize the importance of self-regulated learning and student participation as key behavioral processes associated with digital artifact properties, individual innovativeness, learning continuance, and learner performance. This provides a more process-oriented understanding of the associations between digital technologies and academic development after adoption has occurred.

Third, the study provides empirical support for conceptualizing digital artifacts through the properties of design, elaborateness, and semantic ability. By demonstrating the significant relationships between these properties and learner behaviors, the study highlights the importance of examining the characteristics of digital artifacts themselves rather than focusing exclusively on learners’ perceptions of usefulness or ease of use.

Finally, the study extends the application of the theory of semiotic mediation to contemporary digital learning environments. The findings suggest that digital artifacts, including AI-enabled tools, can function as mediating resources whose properties are associated with how learners regulate, participate in, and continue their learning. This provides further theoretical support for applying semiotic mediation to understand learning in increasingly technology-rich educational contexts.

### 6.2. Implications for Practice

The findings of this study offer several practical implications for educators, instructional designers, and academic administrators seeking to enhance learner performance in digital learning environments. First, the results highlight the importance of providing learners with opportunities to use digital artifacts independently and purposefully within self-regulated learning activities. Educational environments should therefore support learners not only in accessing digital tools but also in exploring, adapting, and using them in ways that align with their individual learning needs.

Second, the implication for instructional designers includes moving beyond simply making digital artifacts visually attractive and instead developing them in a way that supports students’ self-regulated learning processes. In practice, elaborateness needed to be operationalized through multi-layered information structures. Such structures need to provide a core concept first, followed by examples, additional explanations, and reflective prompts. Such elaborateness can allow students to progressively use artifacts for their educational purposes and can also enable greater engagement. Further, this kind of elaborateness will also make the design more effective while simultaneously enhancing semantic ability independently of their elaborateness. From both the design and semantic ability perspectives, these digital artifacts can incorporate self-monitoring elements, such as guiding questions, progress indicators, short knowledge checks, and prompts encouraging students to reflect on their understanding. These features can help students plan, monitor, and evaluate their own learning while interacting with digital content. However, it is important to keep in mind that the core purpose of such artifacts should be to improve meaningful engagement and facilitate self-regulated learning. Thus, by making digital artifacts meaningful, understandable, and engaging, institutions can support sustained learner engagement and potentially strengthen learning continuance and subsequent performance.

Third, the findings suggest that attention should extend beyond technology adoption to the quality of learners’ post-use experiences. Since many students already engage regularly with digital technologies, including AI-enabled tools, educators should focus on how the design, elaborateness, and semantic clarity of these resources support participation, experimentation, and sustained engagement. Digital learning activities should therefore be structured to encourage active interaction rather than passive consumption.

Fourth, curriculum and learning activities can be designed to combine instructor-guided learning with opportunities for independent exploration using digital artifacts. Core concepts may be introduced through formal instruction, while complementary activities can encourage students to regulate their own learning, participate actively, and experiment with digital resources. Such an approach may help support innovativeness, learning continuance, and learner performance.

Finally, institutions should consider the pedagogical properties of digital and AI-enabled tools when integrating them into educational practice. Selecting or designing technologies with clear structure, rich representations, and meaningful semantic organization may increase their potential to support effective learning behaviors and outcomes.

### 6.3. Limitations

The present study has several limitations that should be acknowledged. First, the data were collected from a limited number of universities in the Kingdom of Saudi Arabia. Although the sample included students from different academic disciplines and educational levels, the geographic and institutional scope may limit the generalizability of the findings to other educational and cultural contexts.

Second, the study employed a cross-sectional survey design, which limits the ability to draw causal conclusions about the relationships among digital artifact properties, learner behaviors, and performance outcomes. Future longitudinal or experimental studies could provide evidence regarding how these relationships develop over time.

Third, all study variables were measured using self-reported survey data. Although Harman’s single-factor test suggested that common method bias was not a substantial concern, this procedure provides only a basic diagnostic and cannot completely rule out the presence of common method variance. Future studies could therefore employ additional procedural and statistical remedies and combine self-reported measures with behavioral or performance-based data.

Fourth, the proposed model does not capture all psychological and cognitive factors that may be associated with digital artifacts. Variables such as enjoyment, motivation, perceived cognitive load, and emotional responses may further explain how learners interact with digital and AI-enabled tools. Including such constructs in future models could provide a more comprehensive understanding underlying academic development. Consistently, our research has drawn the properties of digital artifacts from the semiotic mediation. However, theory also argues for other key variables that were not yet included in the current study. These include factors such as editability, interactivity, reprogrammability, and distributability. Thus, future research could extend the current debate by examining these constructs more systematically and exploring their distinct contributions to learning and technology use.

Fifth, our study is also subject to limitations regarding the heterogeneity of participants’ experiences with digital artifacts. Although participants had experience using, designing, or modifying digital artifacts, the study did not fully account for differences in the frequency, duration, instructional setting, disciplinary context, or specific types of artifacts used. Such variation may be related to learners’ perceptions and responses, potentially limiting the comparability of their experiences.

Finally, given the variation in respondents’ age and work experience, as well as the possibility that institutional characteristics across the universities sampled in the Kingdom of Saudi Arabia may impact the findings, heterogeneity analyses can be conducted using a single-regression framework. This would allow us to examine whether the estimated relationships differ across age groups, levels of work experience, and types of institutions, consistent with the multiple-regression approach employed in the PLS-SEM analysis.

### 6.4. Future Research Recommendations

Future research can extend the present study in several directions. First, future research should examine the proposed model across more diverse institutional, cultural, and national contexts to assess the generalizability and contextual robustness of the findings. Longitudinal designs with repeated measures of self-regulated learning and learning performance could provide evidence regarding the temporal development of the proposed relationships. In addition, quasi-experimental or randomized designs comparing AI-supported and non-AI learning conditions, including interventions involving different digital artifact designs, could provide more rigorous evidence of causal relationships. Future studies could further adopt mixed-method approaches that combine survey-based measures with observable learning behaviors to examine how exposure to specific forms of digital or AI-enabled support is associated with self-regulated learning, innovativeness, and subsequent learning outcomes.

Second, future studies could incorporate additional psychological and cognitive variables, such as enjoyment, motivation, perceived cognitive load, trust, and emotional responses. Examining these factors may provide a more comprehensive understanding of how learners interact with digital and AI-enabled artifacts and how such interactions are associated with academic development.

Third, future research should distinguish more clearly between conventional digital artifacts and AI-enabled tools. Because the present study considered digital artifacts as a broad category that includes AI-enabled resources, subsequent studies could compare whether the proposed relationships differ across specific technologies, including generative AI systems, intelligent tutoring tools, multimedia resources and other digital learning platforms.

Fourth, researchers could combine self-reported data with behavioral indicators, such as learning analytics, usage logs, task completion patterns, and objective academic performance measures. Such multi-source data would provide evidence regarding the relationships identified in the present model and help reduce concerns associated with common method variance.

Finally, future studies could further investigate the mediating and moderating relationship within the proposed framework. For example, researchers may examine whether prior digital experience, academic discipline, educational level, or frequency of AI use changes the strength of the relationships among artifact properties, self-regulated learning, participation, innovativeness, continuance, and learner performance.

## Figures and Tables

**Table 1:** Survey instrument.

S.No.	Variables	No. of Items	Source
1	Digital Artifact Design	10	(Blijlevens et al., 2017)
2	Digital Artifact Elaborateness	12	(Reynolds, 1997)
3	Digital Artifact Semantic Ability	5	(DeLone & McLean, 2003)
4	Self-Regulated Learning	12	(Erdogan & Senemoglu, 2016)
5	Student Participation	12	(Anderson et al., 2019)
6	Individual Innovativeness	10	(Leavitt & Walton, 1975)
7	Learning Continuance	3	(Bhattacherjee, 2001)
8	Learner Performance	11	(Roedel et al., 1994)

**Table 2:** Total variance explained (Harman’s single-factor test).

Factor	Total	% of Variance	Cumulative %	Total	% of Variance	Cumulative %
1	21.722	28.582	28.582	21.037	27.680	27.680
2	9.611	12.646	41.227			
3	5.647	7.430	48.657			
4	4.875	6.414	55.071			
5	4.586	6.034	61.106			

Extraction Method: Principal Axis Factoring. Columns 2–4 report initial eigenvalues; columns 5–7 report extraction sums of squared loadings (first factor only).

**Table 3:** Descriptive analysis of the sample.

Item	%
Gender	
Male	64%
Female	36%
Age	
18–24	68.3%
25–34	27.1%
35–44	4.6%
Education level	
Completed High School	5.7%
Undergraduate	51.7%
Post-Graduate	34.3%
Completed or Enrolled in Doctoral Studies	8.3%
Work experience	
0–4 Years	73.1%
5–10 Years	20.0%
11 and More Years	6.9%
Field of Education	
Business Administration	26.3%
Engineering and Technology	23.7%
Medical and Related Science	16.9%
Social Science and Humanities	17.1%
Data Science and Artificial Intelligence	16.0%

**Table 4:** Construct reliability and validity.

Construct	Cronbach’s Alpha	Composite Reliability	AVE
Digital Artifact Design	0.960	0.965	0.734
Digital Artifact Elaborateness	0.972	0.975	0.765
Digital Artifact Semantic Ability	0.932	0.949	0.787
Individual Innovativeness	0.926	0.938	0.602
Learning Continuance	0.822	0.894	0.738
Learner Performance	0.930	0.940	0.590
Self-Regulated Learning	0.948	0.954	0.635
Student Participation	0.948	0.955	0.637

**Table 5:** Discriminant validity using the Heterotrait–Monotrait ratio (HTMT).

	DD	DE	DAS	INN	LC	P	SRL
Digital Artifact Elaborateness (EL)	0.094						
Digital Artifact Semantic Ability (DAS)	0.049	0.035					
Individual Innovativeness (INN)	0.356	0.275	0.345				
Learning Continuance (LC)	0.279	0.144	0.179	0.569			
Learner Performance (P)	0.262	0.208	0.177	0.667	0.651		
Self-Regulated Learning (SRL)	0.434	0.316	0.355	0.615	0.329	0.414	
Student Participation (PN)	0.348	0.378	0.375	0.623	0.338	0.393	0.404

**Table 6:** R-square and R-square adjusted.

Construct	R-Square	R-Square Adjusted
Individual Innovativeness	0.488	0.485
Learning Continuance	0.247	0.245
Learner Performance	0.477	0.474
Self-Regulated Learning	0.390	0.385
Student Participation	0.382	0.376

**Table 7:** Standardized Root Mean Square Residual (SRMR).

	Saturated Model	Estimated Model
SRMR	0.036	0.038

**Table 8:** Assessment of Effect size.

	Self-Regulated Learning	Student Participations	Individual Innovativeness	Learning Continuance	Learning Performance
Digital Artifact Design	0.308	0.207			
Digital Artifact Elaborateness	0.189	0.247			
Digital Artifact Semantic Ability	0.162	0.181			
Individual Innovativeness				0.328	0.290
Learning Continuance					0.174
Learning Performance					
Self-Regulated Learning			0.284		
Student Participations			0.303		

**Table 9:** Structural model path coefficients.

Path	Original Sample (O)	Standard Deviation	T Statistics	*p* Values
Digital Artifact Design → Self-Regulated Learning	0.435	0.038	11.565	<0.001
Digital Artifact Design → Student Participation	0.359	0.047	7.695	<0.001
Digital Artifact Elaborateness → Self-Regulated Learning	0.341	0.042	8.113	<0.001
Digital Artifact Elaborateness → Student Participation	0.393	0.039	10.016	<0.001
Digital Artifact Semantic Ability → Self-Regulated Learning	0.315	0.042	7.450	<0.001
Digital Artifact Semantic Ability → Student Participation	0.335	0.038	8.775	<0.001
Individual Innovativeness → Learning Continuance	0.497	0.036	13.968	<0.001
Individual Innovativeness → Learner Performance	0.449	0.041	11.067	<0.001
Learning Continuance → Learner Performance	0.348	0.041	8.521	<0.001
Self-Regulated Learning → Individual Innovativeness	0.413	0.038	10.829	<0.001
Student Participation → Individual Innovativeness	0.427	0.037	11.607	<0.001

**Table 10:** Mediation (indirect effect) analysis.

Path	Original Sample (O)	Standard Deviation	T Statistics	*p* Values
Self-Regulated Learning → Individual Innovativeness → Learning Continuance	0.205	0.025	8.279	<0.001
Self-Regulated Learning → Individual Innovativeness → Learner Performance	0.185	0.024	7.613	<0.001
Student Participation → Individual Innovativeness → Learning Continuance	0.212	0.025	8.532	<0.001
Student Participation → Individual Innovativeness → Learner Performance	0.192	0.024	7.959	<0.001
Digital Artifact Design → Self-Regulated Learning → Individual Innovativeness	0.180	0.024	7.580	<0.001
Digital Artifact Elaborateness → Self-Regulated Learning → Individual Innovativeness	0.141	0.021	6.652	<0.001
Digital Artifact Semantic Ability → Self-Regulated Learning → Individual Innovativeness	0.130	0.021	6.140	<0.001
Individual Innovativeness → Learning Continuance → Learner Performance	0.173	0.023	7.475	<0.001
Digital Artifact Design → Student Participation → Individual Innovativeness	0.153	0.026	5.911	<0.001
Digital Artifact Elaborateness → Student Participation → Individual Innovativeness	0.168	0.021	8.115	<0.001
Digital Artifact Semantic Ability → Student Participation → Individual Innovativeness	0.143	0.020	7.009	<0.001
Digital Artifact Design → Student Participations → Individual Innovativeness → Learning Continuance → Learning Performance	0.026	4.486	0.006	0.000
Digital Artifact Elaborateness → Self-Regulated Learning → Individual Innovativeness → Learning Performance	0.063	5.733	0.011	0.000
Digital Artifact Semantic Ability → Self-Regulated Learning → Individual Innovativeness → Learning Continuance → Learning Performance	0.022	4.744	0.005	0.000

## Data Availability

The original contributions presented in this study are included in the article. Further inquiries can be directed to the corresponding author.

## Appendix A

**Table A1:** Items of the measurement instrument.

S.No.	Item
1.	Digital Artifact Design
a.	The design of educational digital artifacts, such as infographics, videos, and digital media illustrations, is familiar to me.
b.	The design of educational digital artifacts, such as infographics, videos, and digital media illustrations, follows clear and consistent standards.
c.	The design of educational digital artifacts, such as infographics, videos, and digital media illustrations, is original and distinctive.
d.	The design of educational digital artifacts, such as infographics, videos, and digital media illustrations, is innovative and creative.
e.	The design of educational digital artifacts, such as infographics, videos, and digital media illustrations, integrates its different components effectively.
f.	The design of educational digital artifacts, such as infographics, videos, and digital media illustrations, is well structured and organized.
g.	The design of educational digital artifacts, such as infographics, videos, and digital media illustrations, is coherent and consistent.
h.	The design of educational digital artifacts, such as infographics, videos, and digital media illustrations, consists of well-connected and interrelated components.
i.	The design of educational digital artifacts, such as infographics, videos, and digital media illustrations, demonstrates visual variety and diversity.
j.	The design of educational digital artifacts, such as infographics, videos, and digital media illustrations, integrates a broad range of relevant components.
2.	Digital Artifact Elaborateness
a.	The digital artifact encourages deep analysis of its content.
b.	The artifact effectively captures and sustains my attention.
c.	The artifact stimulates deep thinking about its message.
d.	The artifact engages me intellectually with its ideas.
e.	The artifact requires meaningful cognitive effort to understand.
f.	The artifact helps me stay focused on its central ideas.
g.	The artifact encourages me to think carefully about its content.
h.	The artifact makes me reflect on the implications of its ideas.
i.	The artifact stimulates mental engagement rather than passive viewing.
j.	The artifact encourages exploration of related ideas beyond the content.
k.	The artifact presents information in a way that is mentally engaging.
l.	The artifact provides a rich and thought-provoking experience.
3.	Digital Artifact Semantic Ability
a.	Digital artifacts, such as infographics/videos/digital media illustrations, provide all the necessary information to understand the message completely.
b.	Digital artifacts, such as infographics/videos/digital media illustrations, present the content in a clear and easy-to-understand manner.
c.	Digital artifacts, such as infographics/videos/digital media illustrations, are relevant to my needs or interests.
d.	Digital artifacts, such as infographics/videos/digital media illustrations, present information that is meaningful and aligned with the topic.
e.	Digital artifacts, such as infographics/videos/digital media illustrations, convey trustworthy and reliable information.
4.	Self-Regulated Learning
a.	I usually study in an environment where I can concentrate effectively.
b.	I prepare a weekly to-do list to organize my learning tasks.
c.	I complete my assignments when I know my instructor will review them.
d.	I identify the key points in the material and draw connections between them.
e.	I review additional sources after class.
f.	When I do not understand something, I seek assistance from a peer or an instructor.
g.	I try to find the easiest way of completing my assignments.
h.	While reading academic material or reviewing my notes, I sometimes stop and ask myself, “Do I understand the point here?”
i.	I explain the topic I am studying to another person.
j.	Generally, I do not revise assignments after I have completed them.
k.	I promise to reward myself after I receive a good grade on an exam or assignment.
l.	When I experience failure, I feel discouraged but do not take action to improve the situation.
5.	Student Participation
a.	At my university, students work with instructors outside of class time to contribute to university activities and initiatives.
b.	At my university, students collaborate outside of class time to contribute to improvements in university practices.
c.	In university activities such as student clubs, events, field trips, volunteering, and social activities, my classmates and I often make decisions together.
d.	In class, my classmates and I often make decisions together about our learning.
e.	In class, my instructors and I often make decisions together about my learning.
f.	At my university, I usually get to express my views about how my work is assessed.
g.	At my university, I usually get to express my views about how I am taught.
h.	At my university, I usually get to express my views about what I learn.
i.	At my university, I usually get to express my views about class rules and expectations.
j.	At my university, I usually get to express my views about assignments and coursework.
k.	In university activities, I usually get to express my views about how the activities are organized.
l.	In university activities, I usually get to express my views about which activities are offered.
6.	Individual Innovativeness
a.	I like trying new ways of learning even if they are different from usual teaching methods.
b.	I enjoy experimenting with new ideas in my academic work.
c.	I am willing to take risks when trying new learning approaches.
d.	I often suggest new ideas to improve class activities.
e.	I like exploring alternative solutions to academic problems.
f.	I actively look for new methods to complete my assignments.
g.	I enjoy working on tasks that require creativity and originality.
h.	I am open to using new technologies for learning.
i.	I prefer doing things in new and different ways rather than following routine methods.
j.	I like participating in activities that involve innovation and new ideas.
7.	Learning Continuance
a.	I intend to continue learning using various digital artifacts and tools in the future.
b.	My intention is to continue using various digital artifacts and tools rather than switching to traditional learning methods.
c.	If possible, I would like to keep using various digital artifacts and tools regularly.
8.	Learner Performance
a.	I enjoy challenging academic assignments.
b.	I increase my effort after experiencing an academic setback.
c.	I adapt effectively to challenging academic circumstances.
d.	I am highly determined to achieve my academic goals.
e.	Developing a strong mastery of academic subjects is important to me.
f.	I am intrinsically motivated to learn.
g.	I feel most satisfied when I work hard to achieve an academic goal.
h.	It is important for me to achieve strong academic results compared with my peers.
i.	Academic success largely depends on sustained effort.
j.	Persisting with a challenging academic task is rewarding.
k.	Every university student has the potential to become a successful learner.

## Appendix B

**Table A2:** Variance inflation factor (VIF) values

Constructs	Items	VIF
Self-Regulated Learning	SRL_1	2.556
	SRL_2	2.205
	SRL_3	2.434
	SRL_4	2.570
	SRL_5	2.361
	SRL_6	2.294
	SRL_7	2.943
	SRL_8	2.298
	SRL_9	2.229
	SRL_10	2.437
	SRL_11	2.236
	SRL_12	2.400
Learning continuance	conti_1	1.808
	conti_2	1.910
	conti_3	1.826
Digital artifact Design	design_1	2.979
	design_2	2.795
	design_3	3.429
	design_4	3.043
	design_5	3.097
	design_6	3.255
	design_7	3.240
	design_8	3.126
	design_9	3.074
	design_10	3.197
Digital artifact Elaborateness	elab_1	3.479
	elab_2	3.940
	elab_3	3.581
	elab_4	4.018
	elab_5	3.463
	elab_6	3.539
	elab_7	3.953
	elab_8	4.025
	elab_9	3.657
	elab_10	3.343
	elab_11	4.078
	elab_12	3.745
Innovativeness	innov_1	2.091
	innov_2	2.064
	innov_3	2.371
	innov_4	2.218
	innov_5	2.168
	innov_6	1.981
	innov_7	1.889
	innov_8	2.291
	innov_9	2.075
	innov_10	2.132
Student Participations	part_1	2.579
	part_2	2.353
	part_3	2.510
	part_4	2.181
	part_5	2.491
	part_6	2.426
	part_7	2.341
	part_8	2.601
	part_9	2.551
	part_10	2.168
	part_11	2.439
	part_12	2.368
Learner Performance	perf_1	2.040
	perf_2	2.095
	perf_3	2.296
	perf_4	1.974
	perf_5	2.139
	perf_6	2.005
	perf_7	2.051
	perf_8	2.040
	perf_9	1.987
	perf_10	2.234
	perf_11	2.050
Digital artifact Semantic Ability	sem_1	3.060
	sem_2	3.103
	sem_3	3.066
	sem_4	3.050
	sem_5	3.181

## Appendix C. Full List of Indirect Relationships

**Table A3:** Indirect Relationships.

Specific Indirect Path	Co-Efficient	Standard Deviation	T Statistics	*p* Values
Digital Artifact Design → Student Participations → Individual Innovativeness → Learning Continuance → Learning Performance	0.026	0.006	4.486	0.000
Digital Artifact Elaborateness → Self-Regulated Learning → Individual Innovativeness → Learning Performance	0.063	0.011	5.733	0.000
Digital Artifact Elaborateness → Student Participations → Individual Innovativeness → Learning Continuance	0.083	0.012	6.760	0.000
Self-Regulated Learning → Individual Innovativeness → Learning Continuance	0.205	0.025	8.279	0.000
Digital Artifact Elaborateness → Student Participations → Individual Innovativeness → Learning Performance	0.075	0.012	6.439	0.000
Self-Regulated Learning → Individual Innovativeness → Learning Performance	0.185	0.024	7.613	0.000
Student Participations → Individual Innovativeness → Learning Continuance	0.212	0.025	8.532	0.000
Student Participations → Individual Innovativeness → Learning Performance	0.192	0.024	7.959	0.000
Digital Artifact Elaborateness → Self-Regulated Learning → Individual Innovativeness → Learning Continuance	0.070	0.012	5.885	0.000
Digital Artifact Semantic Ability → Self-Regulated Learning → Individual Innovativeness → Learning Continuance → Learning Performance	0.022	0.005	4.744	0.000
Student Participations → Individual Innovativeness → Learning Continuance → Learning Performance	0.074	0.012	6.133	0.000
Digital Artifact Elaborateness → Self-Regulated Learning → Individual Innovativeness → Learning Continuance → Learning Performance	0.024	0.005	4.845	0.000
Digital Artifact Design → Self-Regulated Learning → Individual Innovativeness	0.180	0.024	7.580	0.000
Digital Artifact Elaborateness → Self-Regulated Learning → Individual Innovativeness	0.141	0.021	6.652	0.000
Digital Artifact Semantic Ability → Self-Regulated Learning → Individual Innovativeness	0.130	0.021	6.140	0.000
Individual Innovativeness → Learning Continuance → Learning Performance	0.173	0.023	7.475	0.000
Digital Artifact Design → Student Participations → Individual Innovativeness → Learning Performance	0.069	0.013	5.141	0.000
Digital Artifact Design → Self-Regulated Learning → Individual Innovativeness → Learning Continuance	0.089	0.014	6.356	0.000
Digital Artifact Design → Self-Regulated Learning → Individual Innovativeness → Learning Performance	0.081	0.013	6.046	0.000
Digital Artifact Design → Student Participations → Individual Innovativeness → Learning Continuance	0.076	0.015	5.216	0.000
Digital Artifact Semantic Ability → Student Participations → Individual Innovativeness → Learning Performance	0.064	0.011	6.030	0.000
Digital Artifact Semantic Ability → Self-Regulated Learning → Individual Innovativeness → Learning Performance	0.058	0.011	5.242	0.000
Digital Artifact Semantic Ability → Student Participations → Individual Innovativeness → Learning Continuance	0.071	0.012	6.153	0.000
Digital Artifact Semantic Ability → Self-Regulated Learning → Individual Innovativeness → Learning Continuance	0.065	0.012	5.593	0.000
Digital Artifact Design → Self-Regulated Learning → Individual Innovativeness → Learning Continuance → Learning Performance	0.031	0.006	5.174	0.000
Digital Artifact Semantic Ability → Student Participations → Individual Innovativeness → Learning Continuance → Learning Performance	0.025	0.005	4.932	0.000
Self-Regulated Learning → Individual Innovativeness → Learning Continuance → Learning Performance	0.071	0.012	6.071	0.000
Digital Artifact Design → Student Participations → Individual Innovativeness	0.153	0.026	5.911	0.000
Digital Artifact Elaborateness → Student Participations → Individual Innovativeness → Learning Continuance → Learning Performance	0.029	0.005	5.338	0.000
Digital Artifact Elaborateness → Student Participations → Individual Innovativeness	0.168	0.021	8.115	0.000
Digital Artifact Semantic Ability → Student Participations → Individual Innovativeness	0.143	0.020	7.009	0.000
